# Histological grade, elastosis, DNA ploidy and the response to chemotherapy of breast cancer.

**DOI:** 10.1038/bjc.1987.89

**Published:** 1987-04

**Authors:** J. R. Masters, R. S. Camplejohn, R. R. Millis, R. D. Rubens

## Abstract

The relationships between response to chemotherapy of advanced breast cancer and the histological type, grade, elastosis content and DNA ploidy of the primary tumours were examined using paraffin-embedded tissue derived from 125 patients. Higher response rates were seen amongst tumours with a high elastosis content and those that were diploid. However, selection of patients with advanced breast cancer for chemotherapy will not be assisted significantly by an assessment of these features in the primary tumour.


Br. J. Cancer (1987), 55, 455–457

© The Macmillan Press Ltd., 1987

### Histological grade, elastosis, DNA ploidy and the response to chemotherapy of breast cancer

J.R.W. Masters[1], R.S. Camplejohn[2], R.R. Millis[3] & R.D. Rubens[3]

[1]*Department of Histopathology, Institute of Urology, St. Paul's Hospital, 24 Endell Street, London, WC2H 9AE;* [2]*Richard Dimbleby Department of Cancer Research, United Medical and Dental Schools, St. Thomas' Hospital, London, SE1 7EH; and* [3]*Imperial Cancer Research Fund Clinical Oncology Unit, United Medical and Dental Schools, Guy's Hospital, London Bridge, London, SE1 9RT, UK.*

**Summary** The relationships between response to chemotherapy of advanced breast cancer and the histological type, grade, elastosis content and DNA ploidy of the primary tumours were examined using paraffin-embedded tissue derived from 125 patients. Higher response rates were seen amongst tumours with a high elastosis content and those that were diploid. However, selection of patients with advanced breast cancer for chemotherapy will not be assisted significantly by an assessment of these features in the primary tumour.

Objective regression of advanced breast cancer can be achieved in approximately 50% of patients treated by chemotherapy, but there is no reliable method of predicting which patients will benefit. In previous studies we demonstrated an association between certain pathological features and response to endocrine therapy. Patients with well-differentiated carcinomas or those containing abundant elastosis showed a significantly higher frequency of response (Millis *et al.*, 1981; Masters *et al.*, 1986). In addition, in some studies, DNA ploidy is associated with oestrogen receptor status and with histological differentiation (see review by Friedlander *et al.*, 1984) and might provide an objective and reproducible alternative to histological grading. The purpose of this study was to determine whether histological grade, elastosis and DNA ploidy are related to response to chemotherapy.

### Materials and methods

One hundred and twenty-five patients were studied. The criteria for eligibility were (a) no prior chemotherapy and (b) availability of sections from the primary tumour for histological evaluation. Response to the first chemotherapy regimen for advanced disease, either adriamycin alone or with vincristine (Steiner *et al.*, 1983) was assessed according to UICC criteria (Hayward *et al.*, 1977) as objective regression (complete or partial response) or no response (no change or progressive disease). Histological grade was assessed in infiltrating ductal tumours using the classification of Bloom and Richardson (1957), as grade 1 (well differentiated), grade 2 (moderately differentiated) or grade 3 (poorly differentiated). Fifteen lobular tumours were not graded. Elastosis was demonstrated using orcein stained sections, available from 106 cases, and assessed as previously described (Masters *et al.*, 1979), and classified as level 2 (abundant), level 1 (present) or level 0 (absent). DNA flow cytometry was performed on cell suspensions prepared from formalin fixed paraffin blocks, available in 86 cases. The suspensions were preparing using the method described by Hedley *et al.* (1983) with minor modifications (Camplejohn & Macartney, 1985). Briefly, 40 $\mu$m sections (usually one section per case) were cut, dewaxed in xylene and rehydrated through a series of alcohols into water. The sections were then treated for 30 min at 37°C in a 5 mg ml$^{-1}$ solution of pepsin (Sigma, Dorset, UK) at pH 1.5. The resulting suspension was spun at 2,000 rpm for 3 min and resuspended in 2.5 ml of Isoton (Coulter Electronics, Luton, UK) containing 1 $\mu$g ml$^{-1}$ DAPI (4′,6-diamidino,-2 phenylindol dihydrochloride, Boehringer, West Germany). The suspensions were passed through a 25 gauge needle before filtration through 35 $\mu$m pore size polyester gauze. Samples were analysed with a Becton Dickinson FACS Analyser (see Camplejohn & Macartney, 1985). To construct each histogram at least 10,000 cells were scanned and the results stored on disc for further study. The DNA index was determined for each case. The DNA index is calculated by measuring the position of any aneuploid G1 peak relative to the normal G0/G1 peak. A DNA index of 1.0 indicates the presence of only diploid cells. S-phase fractions were calculated by the method of Baisch *et al.*, (1975). For DNA aneuploid tumours the S-phase fraction of DNA aneuploid tumour cells was calculated and expressed as a percentage of total DNA aneuploid cells. There are well-recognised problems in the calculation of S-phase fractions in both DNA diploid and aneuploid tissues, and caution must be exercised in interpreting such data. Despite these limitations, many published studies have shown that the values generated can have biological and clinical significance. For example, in non-Hodgkin's lymphoma S-phase estimates calculated in this manner are associated with prognosis (Macartney *et al.*, 1986) and in breast cancer are associated with epidermal growth factor expression and tumour grade (Walker & Camplejohn, 1986). For tumours with multiple DNA aneuploid populations, no S-phase calculations were attempted. A full peak coefficient of variation for the G0/G1 peak was calculated for each sample using software supplied by Becton Dickinson.

### Results

Objective response was seen in 56 of the 125 patients (45%). There was no significant difference in the response rate of the lobular (47%) and the histological grade 2 (49%) and 3 (42%) ductal carcinomas. However, none of the 4 well differentiated grade 1 ductal tumours responded (Table I). Tumours with abundant elastosis were more likely to respond (100%) than those with some (49%) or no (29%) elastosis; $P<0.01$, chi-squared test with 2 degrees of freedom (Table I).

DNA ploidy was assessable (coefficient of variation range 3.0–7.7, mean 5.1) in 76/86 (88%) of the tumours studied, 16 (21%) of which were diploid and 60 (79%) DNA aneuploid. Response to chemotherapy was observed in 11/16 (69%) diploid compared with 27/60 (45%) DNA aneuploid tumours (chi-squared with Yates' correction = 1.98, not significant at 5% level). The proportion of DNA aneuploid cells within each tumour was classified as low (<30%), moderate (30–60%) or high (>60%). Response to chemotherapy was observed in 9/17 (53%) of the tumours with a low fraction

Correspondence: J.R.W. Masters.
Received 18 July 1986; and in revised form 17 November 1986.

**Table I** Summary of the number and proportion of patients responding to each form of chemotherapy, subdivided according to the features studied

| | *Treatment outcome* | | |
|---|---|---|---|
| | *No response* | *Regression* | *%Regression* |
| *Therapy* | | | |
| Adriamycin | 30 | 25 | 25/55(45%) |
| Adriamycin + vincristine | 39 | 31 | 31/70(44%) |
| *Tumour grade* | | | |
| Lobular | 8 | 7 | 7/15(47%) |
| 1 | 4 | 0 | 0/4 (0%) |
| 2 | 32 | 31 | 31/63(49%) |
| 3 | 25 | 18 | 18/43(42%) |
| *Elastosis* | | | |
| 0 | 17 | 7 | 7/24(29%) |
| 1 | 38 | 37 | 37/75(49%) |
| 2 | 0 | 7 | 7/7 (100%) |
| *DNA ploidy* | | | |
| Diploid | 5 | 11 | 11/16(69%) |
| Aneuploid | 33 | 27 | 27/60(45%) |
| *%DNA aneuploid cells* | | | |
| Low | 8 | 9 | 9/17(53%) |
| Medium | 18 | 13 | 13/31(42%) |
| High | 4 | 5 | 5/9 (56%) |
| *%S-phase cells* | | | |
| Low | 6 | 8 | 8/14(57%) |
| Medium | 16 | 17 | 17/33(52%) |
| High | 10 | 12 | 12/22(55%) |

**Table II** Summary of the number of each histological grade of tumour, subdivided according to their elastosis content, DNA ploidy and percentages of DNA aneuploid and S-phase cells

| | | *Tumour grade* | | | |
|---|---|---|---|---|---|
| | *Lobular* | *1* | *2* | *3* | *%Grade 3* |
| *Elastosis* | | | | | |
| 0 | 2 | 1 | 12 | 9 | 9/22(41%) |
| 1 | 8 | 3 | 36 | 28 | 28/67(42%) |
| 2 | 1 | 0 | 4 | 2 | 2/6 (33%) |
| *DNA ploidy* | | | | | |
| Diploid | 4 | 0 | 11 | 1 | 1/12 (8%) |
| Aneuploid | 2 | 2 | 24 | 32 | 32/58(55%) |
| *%DNA aneuploid cells* | | | | | |
| Low | 1 | 1 | 9 | 6 | 6/16(38%) |
| Medium | 1 | 1 | 11 | 18 | 18/30(60%) |
| High | 0 | 0 | 2 | 7 | 7/9 (78%) |
| *%S-phase cells* | | | | | |
| Low | 3 | 1 | 9 | 1 | 1/11 (9%) |
| Medium | 3 | 1 | 16 | 13 | 13/30(43%) |
| High | 0 | 0 | 6 | 16 | 16/22(73%) |

of DNA aneuploid cells, compared with 13/31 (42%) in the moderate category and 5/9 (56%) in the high category. The proportion of S-phase cells in each tumour was classified as low (0–7%), moderate (7.1–14%) or high (greater than 14%). Response to chemotherapy was observed in 8/14 (57%) of the tumours with a low fraction of S-phase cells, compared with 17/33 (52%) of those in the moderate and 12/22 (65%) in the high categories.

Poorly-differentiated grade 3 tumours were more likely (see Table II) to be DNA aneuploid ($P<0.01$, chi-squared test with Yates' correction, comparing grade 3 with grades 1 and 2 combined), have a high proportion of DNA aneuploid cells (chi-squared = 4.59, not significant at 5% level with 2 degrees of freedom comparing the grade 3 tumours with

**Table III** The numbers of tumours containing no, some or abundant elastosis, subdivided according to their DNA ploidy and percentages of DNA aneuploid and S-phase cells

| | *Elastosis* | | | |
|---|---|---|---|---|
| | 0 | 1 | 2 | *%Category 0* |
| *DNA ploidy* | | | | |
| Diploid | 2 | 13 | 1 | 2/16(13%) |
| Aneuploid | 13 | 43 | 4 | 13/60(22%) |
| *%DNA aneuploid cells* | | | | |
| Low | 4 | 11 | 2 | 4/17(24%) |
| Medium | 7 | 21 | 2 | 7/30(23%) |
| High | 1 | 8 | 0 | 1/9 (11%) |
| *%S-phase cells* | | | | |
| Low | 0 | 13 | 1 | 0/14 (0%) |
| Medium | 8 | 22 | 3 | 8/33(24%) |
| High | 5 | 16 | 1 | 5/22(23%) |

grades 1 and 2 combined) and S-phase cells ($P<0.01$, chi-squared test with 2 degrees of freedom comparing grade 3 tumours with grades 1 and 2 combined). However, these features were not associated with the elastosis content of the tumours (Table III). Of the diploid tumours, 10/16 had a low fraction (<7%) of S-phase cells, compared with only 4/53 of the DNA aneuploid tumours ($P<0.001$, chi-squared test with 2 degrees of freedom).

### Discussion

An accurate means of selecting patients who will benefit from chemotherapy is still needed. Associations between response and both thymidine labelling indices and drug sensitivity in the human tumour stem cell assay have been reported, but both these techniques have pratical limitations and are not used routinely. Oestrogen receptor status has no predictive value for response to chemotherapy (Stewart *et al.*, 1984).

In this study we show that histological grade has no predictive value. Elastosis is associated with response, but is of inadequate specificity to assist management. DNA ploidy was studied in a small number of tumours, but again there was no indication that this technique could be applied to the problem of patient selection.

Well-differentiated tumours, with the exceptions of lymphoma and chronic lymphocytic leukaemia, are relatively resistant to chemotherapy (Whitehouse, 1984). None of the four grade I tumours in this study responded to chemotherapy. However, well-differentiated breast tumours tend to be diploid (Friedlander *et al.*, 1984), yet a higher response rate was observed in the diploid tumours. The trend towards better response to chemotherapy in this group of tumours may reflect the greater ability of the more anaplastic tumours to acquire drug resistance, as suggested by Goldie and Coldman (1984). In contrast, there are some studies that indicate that poorly-differentiated tumours are more likely to respond to chemotherapy (Whitehouse, 1984), although this did not apply to the patients in this study. The interrelationships between tumour DNA ploidy, growth rate, fraction of S-phase cells and histological differentiation in relation to response to chemotherapy need further study.

In summary, we have been unable to demonstrate that histological grade, elastosis or DNA ploidy significantly assist the selection of patients with advanced breast cancer for chemotherapy.

We thank Mrs Julie Alder and Mr Michael Stone for their expert technical assistance.

### References

BAISCH, H., GOHDE, W. & LINDEN, W.A. (1975). Analysis of PCP-data to determine the fraction of cells in the various phases of cell cycle. *Radiat. Environ. Biophys.*, **12**, 37.

BLOOM, H.J.G. & RICHARDSON, W.W. (1957). Histologic grading and prognosis in breast cancer. *Br. J. Cancer*, **11**, 359.

CAMPLEJOHN, R.S. & MACARTNEY, J.C. (1985). Comparison of DNA flow cytometry from fresh and paraffin embedded samples of non-Hodgkin's lymphona. *J. Clin. Pathol.*, **38**, 1096.

FRIEDLANDER, M.L., HEDLEY, D.W. & TAYLOR, I.W. (1984). Clinical and biological significance of aneuploidy in human tumours. *J. Clin. Pathol.*, **37**, 961.

GOLDIE, J.H. & COLDMAN, A.J. (1984). The genetic origin of drug resistance in neoplasms: implications for systemic therapy. *Cancer Res.*, **44**, 3643.

HAYWARD, J.L., RUBENS, R.D., CARBONE, P.P., HEUSON, J-C., KUMAOKA, S. & SEGALOFF, A. (1978). Assessment of response to therapy in advanced breast cancer. *Eur. J. Cancer*, **14**, 1291.

HEDLEY, D., FRIEDLANDER, M., TAYLOR, I., RUGG, C. & MUSGROVE, E. (1983). Method for analysis of cellular DNA content of paraffin-embedded pathological material using flow cytometry. *J. Histochem. Cytochem.*, **31**, 1333.

MACARTNEY, J.C., CAMPLEJOHN, R.S., ALDER, J., STONE, M.G. & POWELL, G. (1986). Prognostic importance of DNA flow cytometry in non-Hodgkin's lymphonas. *J. Clin. Pathol.*, **39**, 542.

MASTERS, J.R.W., MILLIS, R.R., KING, R.J.B. & RUBENS, R.D. (1979). Elastosis and response to endocrine therapy in human breast cancer. *Br. J. Cancer*, **39**, 536.

MASTERS, J.R.W., MILLIS, R.R. & RUBENS, R.D. (1986). Response to endocrine therapy and breast cancer differentiation. *Breast Cancer Res. Treat.*, **7**, 31.

MILLIS, R.R., RUBENS, R.D., MASTERS, J.R.W. & MINTON, M.J. (1981). Histological grade and response to endocrine therapy in breast cancer. *Lancet*, **ii**, 101.

STEINER, R., STEWART, J.F., CANTWELL, B.M.J., MINTON, M.J., KNIGHT, R.K. & RUBENS, R.D. (1983). Adriamycin alone or combined with vincristine in the treatment of advanced breast cancer. *Eur. J. Cancer Clin. Oncol.*, **19**, 1553.

STEWART, J.F., HAYWARD, J.L., RUBENS, R.D. & KING, R.J.B. (1984). Oestrogen receptor status of advanced breast cancer immediately before chemotherapy does not predict for response. *Cancer Chemother. Pharmacol.*, **9**, 124.

WALKER, R.A. & CAMPLEJOHN, R.S. (1986). DNA flow cytometry of human breast carcinomas and its relationship to transferrin and epidermal growth factor receptors. *J. Pathol.* (in press).

WHITEHOUSE, J.M. (1984). Clinical setting. In *Antitumor Drug Resistance*, (eds.) Fox, B.W and Fox, M., p. 3. Springer-Verlag: Berlin.


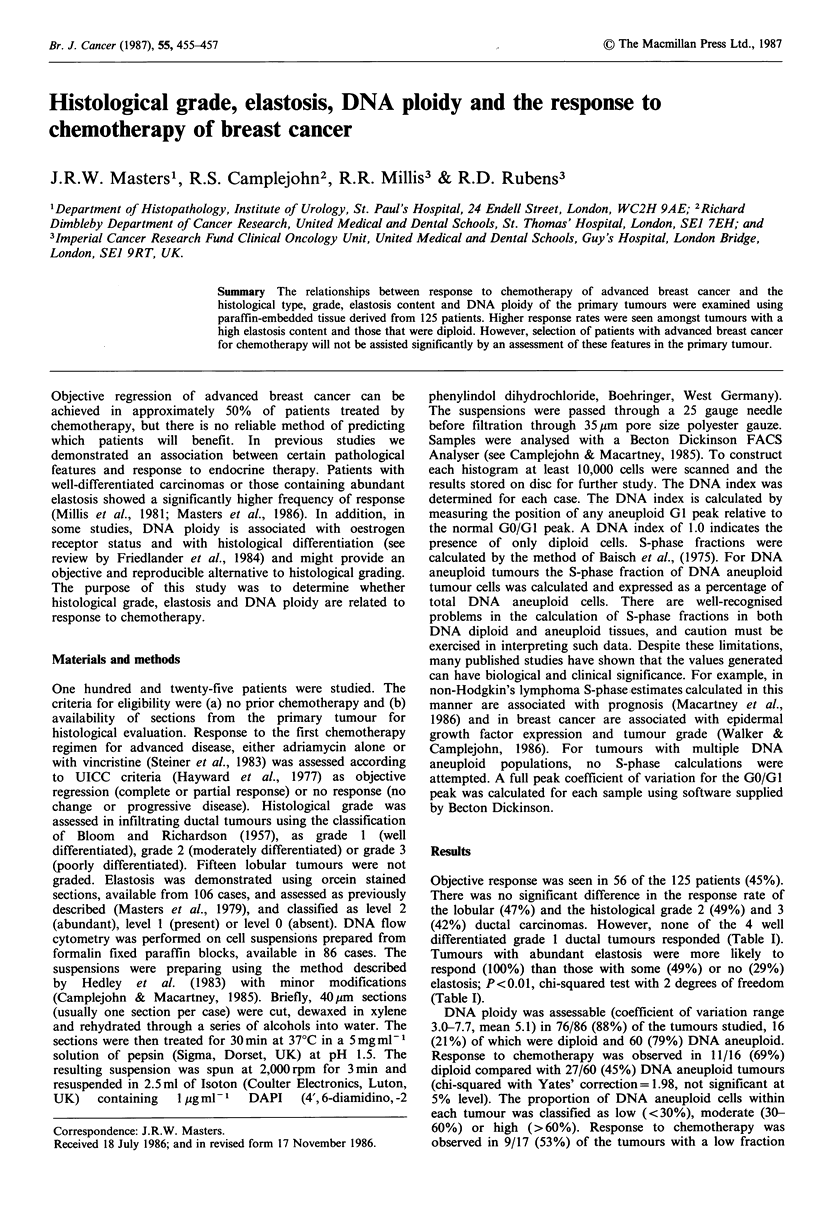

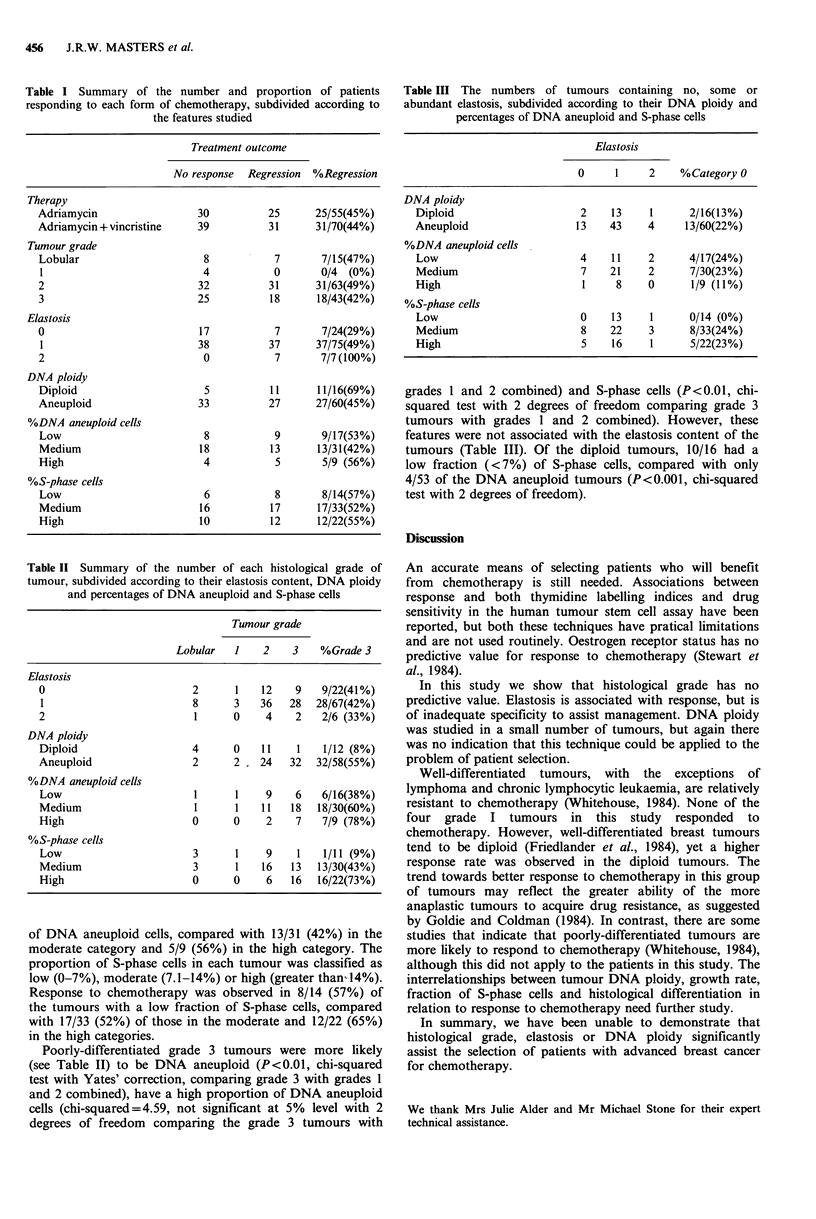

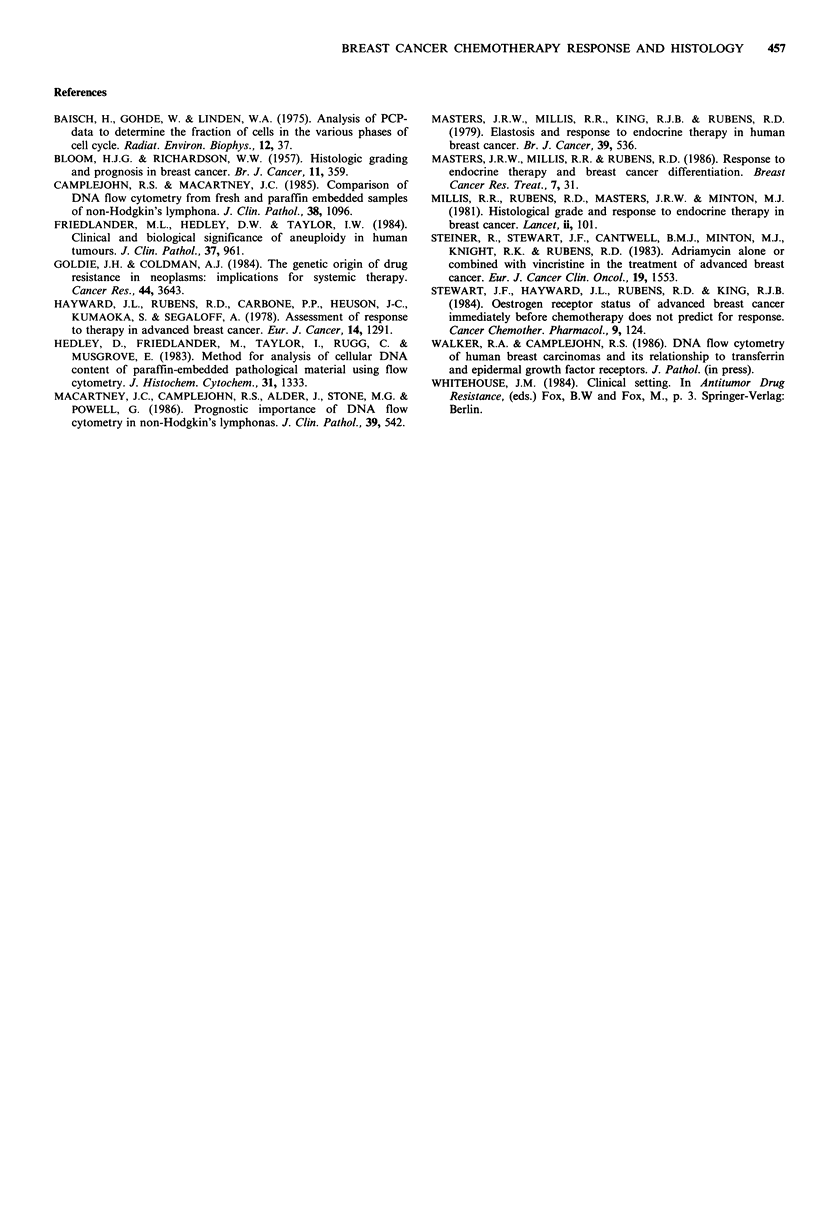


## References

[OCR_00302] BLOOM H. J., RICHARDSON W. W. (1957). Histological grading and prognosis in breast cancer; a study of 1409 cases of which 359 have been followed for 15 years.. Br J Cancer.

[OCR_00306] Camplejohn R. S., Macartney J. C. (1985). Comparison of DNA flow cytometry from fresh and paraffin embedded samples of non-Hodgkin's lymphoma.. J Clin Pathol.

[OCR_00311] Friedlander M. L., Hedley D. W., Taylor I. W. (1984). Clinical and biological significance of aneuploidy in human tumours.. J Clin Pathol.

[OCR_00316] Goldie J. H., Coldman A. J. (1984). The genetic origin of drug resistance in neoplasms: implications for systemic therapy.. Cancer Res.

[OCR_00321] Hayward J. L., Rubens R. D., Carbone P. P., Heuson J. C., Kumaoka S., Segaloff A. (1978). Assessment of response to therapy in advanced breast cancer. A project of the programme on clinical oncology of the International Union against Cancer, Geneva, Switzerland.. Eur J Cancer.

[OCR_00326] Hedley D. W., Friedlander M. L., Taylor I. W., Rugg C. A., Musgrove E. A. (1983). Method for analysis of cellular DNA content of paraffin-embedded pathological material using flow cytometry.. J Histochem Cytochem.

[OCR_00332] Macartney J. C., Camplejohn R. S., Alder J., Stone M. G., Powell G. (1986). Prognostic importance of DNA flow cytometry in non-Hodgkin's lymphomas.. J Clin Pathol.

[OCR_00337] Masters J. R., Millis R. R., King R. J., Rubens R. D. (1979). Elastosis and response to endocrine therapy in human breast cancer.. Br J Cancer.

[OCR_00342] Masters J. R., Millis R. R., Rubens R. D. (1986). Response to endocrine therapy and breast cancer differentiation.. Breast Cancer Res Treat.

[OCR_00347] Millis R. R., Rubens R. D., Masters J. R., Minton M. J. (1981). Histological grade and response to endocrine therapy in breast cancer.. Lancet.

[OCR_00352] Steiner R., Stewart J. F., Cantwell B. M., Minton M. J., Knight R. K., Rubens R. D. (1983). Adriamycin alone or combined with vincristine in the treatment of advanced breast cancer.. Eur J Cancer Clin Oncol.

[OCR_00358] Stewart J. F., Hayward J. L., Rubens R. D., King R. J. (1982). Estrogen receptor status of advanced breast cancer immediately before chemotherapy does not predict for response.. Cancer Chemother Pharmacol.

